# Genomic Position and Markers Associated with the Hull-Less Seed Trait in Pumpkin

**DOI:** 10.3390/plants11091238

**Published:** 2022-05-03

**Authors:** Geoffrey Meru, Yuqing Fu, Swati Shrestha, Vincent Njung’e Michael, Marie Dorval, Riphine Mainviel

**Affiliations:** The Tropical Research and Education Center, Horticultural Sciences Department, University of Florida, 18905 SW 280 ST Homestead, Gainesville, FL 33031, USA; yuqingf@ufl.edu (Y.F.); s.shrestha@ufl.edu (S.S.); michael.vn@ufl.edu (V.N.M.); dorval.m@ufl.edu (M.D.); riphinemainviel@ufl.edu (R.M.)

**Keywords:** QTL-seq, breeding, quantitative trait loci, marker-assisted selection, whole genome resequencing, *Cucurbita pepo*

## Abstract

Pumpkin (*Cucurbita pepo*) seeds are nutritious and valued as a source of vegetable oil, protein, healthy fatty acids, and minerals. Pumpkin seeds that are naturally devoid of the seedcoat (hull-less) are preferred by the industry as they eliminate the need for de-hulling prior to use. A single recessive gene, designated as *n* or *h*, controls the hull-less seed trait in pumpkin. Visual selection for the trait is easy, however, it is resource intensive when applied to large breeding populations. High throughput genotyping assays can aid in the identification of suitable individuals in segregating populations through marker-assisted selection. In the current study, the QTL-seq approach was used to identify genetic loci, SNP markers and candidate genes associated with the hull-less trait in a segregating F_2_ population (*n* = 143) derived from a cross between Kakai (hull-less) × Table Gold Acorn (hulled). The segregation of the hull-less trait in the F_2_ population fit a 3:1 ratio (*p* < 0.05). QTL-seq analysis detected a single QTL on chromosome 12 (*Qtlhull-less-C12*) which was significantly associated with the hull-less trait in *C. pepo*. Twenty-eight SNPs were genotyped in the population, two among which (Ch12_3412046 and Ch12_3417142) were significantly associated (*p* < 0.05) with the hull-less trait in cultivars and accessions of diverse genetic background. Several candidate genes fall within the *Qtlhull-less-C12* interval, among them is the No Apical meristem (NAC) domain-containing protein and a Fiber Protein fb11 gene involved in lignin accumulation and cell wall deposition across plant species, respectively. The findings of this study will facilitate the marker-assisted selection for the hull-less seed trait in pumpkin and further our understanding of the functional mechanisms underlying the trait across cucurbit crops.

## 1. Introduction

Pumpkin (*Cucurbita pepo*) seeds provide an important source of nutrition and income worldwide [1]. In the confectionery industry, pumpkin seed is primarily consumed as a snack in trail mixes or as a functional food-ingredient in nutrition bars, cereal and bread [1,2]. In addition, oil extracted from pumpkin seed is sold for use in the culinary and nutrient-supplement industry [3]. Pumpkin seed is a nutritionally dense food rich in oil (50% *w*/*w*), protein (>35%), healthy unsaturated fatty acids (>85%) and antioxidants [3,4,5,6,7]. These nutritional benefits are associated with a reduced risk of heart related ailments and certain types of cancers [3,5,7,8].

Various Cucurbita species are utilized for pumpkin seed production. In Europe, evidence of the commercial production of pumpkin seed (*C. pepo*) for seed and oil production date back to the seventeenth century in Austria [9]. In North America, cultivars of *C. maxima*, *C. pepo*, and *C. argyrosperma* primarily cultivated for flesh consumption are used to meet the demand for pumpkin seed [1]. Many traits are important in pumpkin seed breeding, key among them being seed yield, seed size, nutrition density and the seed-coat phenotype [5,10]. Seed yield is a function of the harvest index, seed index, seed-yield index and seed size [5,10]. Seed size is important in the confectionery industry where larger seeds are preferred for snacking, but less so for oil production where pumpkin seeds are harvested mechanically [5]. Knowledge of the natural variation in the nutritional content (oil, protein and fatty acid composition) across accessions of Cucurbita is well established and may be explored to develop nutritionally enhanced pumpkin seed cultivars [3,4,5,6,7,10]. The seed-coat phenotype is an important consideration in breeding pumpkin seed cultivars. Pumpkin cultivars with hulled seeds must be mechanically de-hulled prior to use in certain markets, thus adding to production cost. On the contrary, hull-less pumpkin seeds do not require de-hulling prior to use and are preferred by the industry [5].

Originally, the commercial production of pumpkin seed relied on cultivars of *C. pepo* with hulled seeds [11]. However, in the nineteenth century, a spontaneous mutation resulted in cultivars of *C. pepo* devoid of a seed coat (hull-less seed phenotype) in Austria [5,11]. The hull-less seed phenotype in *C. pepo* is controlled by a single recessive allele (designated *n* or *h*) with potential modifiers [11,12]. This allele results in the decreased deposition of lignin and cellulose in the hypodermis, sclerenchyma, and parenchyma tissues of the pumpkin seed coat [4]. Although visual selection for the hull-less seed coat is easy, it is resource intensive when applied to large and complex breeding populations [10]. On the other hand, marker-assisted selection (MAS) for the hull-less seed coat can facilitate the rapid identification of desirable individuals in early generations, thus saving breeding resources [13,14]. The *h* locus in Lady Godiva seed-pumpkin cultivar was mapped as a morphological trait on linkage group LGp9 of *C. pepo*, providing a region for marker design and development [15]. Several simple sequence repeat (SSR) markers adjacent to the locus may be useful in MAS, but have not been validated in diverse genetic backgrounds and require costly capillary-based electrophoresis assays for high-throughput genotyping [16]. Furthermore, no candidate genes are currently described for the hull-less seed coat in *C. pepo* that would facilitate characterization of the mechanisms underlying the trait. The recent availability of a transcriptome for fruit and seed tissue of hull-less (Lady Godiva) and hulled (Sweet REBA) cultivars has provided an important resource for identifying key genes involved in the metabolic pathway for seed-coat formation in *C. pepo* [17].

The QTL-seq approach combines bulk segregant analysis with whole genome re-sequencing to identify single nucleotide polymorphism (SNP) markers tightly linked to a trait of interest [18,19] and has been successfully applied in Cucurbita crops [20,21,22]. The availability of SNP markers is tightly linked to the hull-less seed coat in *C. pepo* coupled to a high throughput genotyping system, such as Kompetitive allele-specific polymerase chain reaction (KASP) [23], would allow for rapid trait introgression in pumpkin breeding programs. The goal of the current study was to employ QTL-seq approach to further characterize the hull-less locus in *C. pepo*, identify candidate genes, and develop and validate high throughput KASP assays for MAS in pumpkin breeding.

## 2. Materials and Methods

### 2.1. Plant Material, Population Development and Phenotyping

An intersubspecific cross was made in the greenhouse between Kakai (hull-less seed phenotype, maternal) and Table Gold Acorn (hulled seed trait, paternal). Kakai (*C. pepo* subspecies pepo) is a semi-vining pumpkin grown for hull-less seeds and ornamental purpose (average fruit weight of 3 kg), while Table Gold Acorn (*C. pepo* subspecies ovifera) is an edible flesh Acorn type squash (average fruit weight of 0.6 kg) (Figure S1). A single F_1_ plant was self-pollinated to yield an F_2_ population (*n* = 143). The parents (*n* = 12, each), F_1_ (*n* = 12) and the F_2_ population were germinated in the greenhouse and transplanted in the field after three weeks at the University of Florida, Tropical Research and Education Center, Homestead, Florida. Standard crop management was implemented following recommended practices for commercial squash production in Florida [24]. A single fruit was generated for each F_2_ individual through self-pollination. At maturity, each fruit was harvested, and seed extracted to determine the seed phenotype (hull-less vs. hulled).

### 2.2. DNA Extraction and Whole Genome Re-Sequencing

Leaf material was collected in liquid nitrogen from each F_2_ individual and stored at −80 °C for future analysis. DNA was extracted from ten hull-less and ten hulled F_2_ individuals and the parents using the FavorPrep Plant DNA kit (Favorgen Biotech Corp., Ping-Tung, Taiwan) according to the manufacturer’s instructions. The concentration of DNA was determined using NanoDrop 8000 Spectrophotometer (Thermo Fisher Scientific, Waltham, MA, USA) and an equal quantity (500 ng) from each individual constituting the bulks were pooled. Pair-end (2 × 150) library construction and whole genome re-sequencing of the two bulks and the parents was performed at the BGI sequencing center (Shenzhen, Guangdong, China) using the Illumina HiSeq X (Illumina, Inc., San Diego, CA, USA).

### 2.3. QTL-Seq Analysis

Parental pair-end sequencing reads were mapped onto the *C. pepo* reference genome [25] using BWA-MEM [26]. A consensus reference fasta-file was generated using SAMtools by replacing *C. pepo* reference alleles with the respective parent alleles across the genome [26,27]. Variant calling between hull-less and hulled bulks was performed by first aligning the reads to the consensus reference sequence followed by removal of duplicate reads and variant calling in GATK [28]. The variants were compiled using GVCFs to obtain a raw variant VCF file, which was used as the raw input data for a QTL analysis in QTLseqr [29]. The runQTLseqAnalysis function was implemented in QTLseqr to detect QTL that was significantly linked with hull-less seed phenotype in *C. pepo*. The SNP-index across all loci was calculated as the proportion of reads that were different from the parental reference allele, while the ∆SNP-index was calculated by determining the difference between SNP-index of hull-less and hulled bulks at each SNP position [19]. Candidate QTL regions were detected using a 1 Mb sliding window in R [30], and the confidence intervals for the ∆SNP-indices were determined using 10,000 simulations.

### 2.4. Marker Test and Candidate Genes

Twenty-eight SNP markers (Table S1) within the identified QTL were converted into KASP (LGC Genomics LLC., Teddington, UK) assays and genotyped in the individuals constituting the hull-less and hulled bulks in the F_2_ population. Significant markers in the bulks were further validated in landraces and commercial cultivars with either the hull-less or hulled seed phenotype, as well as in the entire F_2_ population. Genotyping for KASP markers was performed in 10-μL reactions containing 5 μL of 2x low rox KASP master mix (LGC Genomics LLC., Teddington, UK), 0.16 μL each of forward primers (10 μM), 0.41 μL of reverse primer, 2 μL of genomic DNA (50 ng/μL) and 2.27 μL of H_2_O. The PCR conditions consisted of an initial incubation at 94 °C for 15 min, a touchdown PCR at 94 °C for 20 s, 61 °C for 60 s, with a 0.6 °C decrease per cycle for 10 cycles, followed by 26 cycles of 94 °C for 20 s and 55 °C for 60 s. Fluorescent end-point readings and cluster calling were performed using LightCycler^®^ 480 Instrument II (Roche Life Sciences, Penzberg, Germany). Significant marker-trait association were determined using the Kruskal–Wallis test (*p* ≤ 0.05) in R and confirmed by non-parametric interval mapping in R/qtl [31]. Likelihood of the odds (LOD) values were determined using 4000 permutations and the significance threshold was viewed at 99 percent confidence level. Candidate genes were identified by scanning the QTL interval for homologs involved in lignin and cellulose biosynthesis in plants using the Cucurbit Genomics Database.

## 3. Results

### 3.1. Phenotypic Data

**Figure 1:**
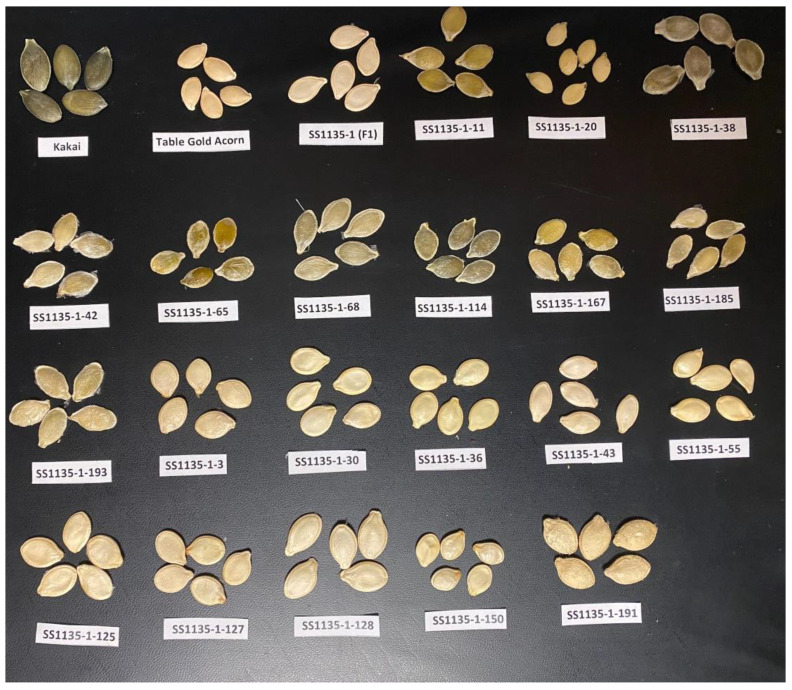
Hull-less and hulled seed phenotype in the parents (Kakai and Table Gold Acorn), the F_1_ (SS1135-1), and segregation in the F_2_ individuals constituting the hull-less (SS1135-1-11, 20, 38, 42, 65, 68, 114, 167, 185 and 193) and hulled (SS1135-1-3, 30, 36, 43, 55, 125, 127, 128, 150 and 191) bulks used for QTLseq analysis.

At fruit maturity, seeds of Kakai exhibited the hull-less phenotype devoid of a seedcoat, while as those of Table Gold Acorn and the F_1_ were of the hulled seed phenotype (Figure 1). Among the 143 F_2_ individuals, 112 and 31 were of the hulled and hull-less seed phenotype, respectively, and were segregated in a phenotypic ratio of 3:1 (*p* < 0.05) (Table 1 and Figure 1).

### 3.2. QTL Analysis

Mapping rate across the genotypes varied from 95.31 to 98.91% with a mean sequencing depth between 57.78 to 70.26 (Table 2).

**Figure 2:**
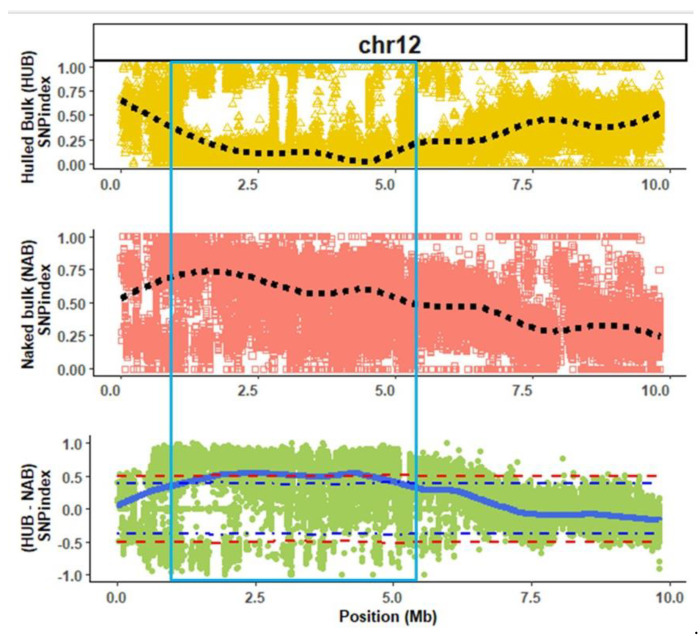
Quantitative trait loci (regions within light-blue square) associated with the hull-less seed phenotype in Kakai cultivar (*Cucurbita pepo*) on chromosome (chr) 12 using Table Gold Acorn as consensus reference genome. The dotted black lines represent smoothed conditional mean of SNP index for hulled (HUB) and hull-less (NAB) bulks, while the solid blue line represents the tricube ∆SNP for the ∆SNP index (HUB SNP index − NAB SNP index). The blue and red dotted lines in the ∆SNP index plot are the 90% and 95% confidence intervals for the regions, respectively.

Alignment of the hull-less and hulled bulks onto the consensus reference genome revealed 1,682,633 and 2,077,247 SNP’s, respectively. QTL-seq analysis detected a single QTL on chromosome 12 (*Qtlhull-less-C12*) significantly associated with the hull-less seed phenotype in *C. pepo* (Figure 2 and Figure S2). *Qtlhull-less-C12* extended from 1.25 Mb to 5.68 Mb on chromosome 12. A similar genomic position for *Qtlhull-less-C12* was identified when Kakai was used as the consensus reference sequence (Figure S3).

### 3.3. Marker Validation and Candidate Genes

Among the twenty eight KASP genotyped in the bulks, twelve were significantly linked (logarithm-of-odds (LOD) > 3) to the hull-less seed phenotype in *C. pepo* (Table 3). Marker-trait associations were further confirmed using non-parametric interval mapping in the F_2_ individuals constituting the bulks (Figure S4). The twelve markers were genotyped and tested for association with the hull-less trait in a set cultivars and landraces of diverse genetic backgrounds. Among the twelve, two SNP markers (Ch12_3412046 and Ch12_3417142) were consistently associated (*p* < 0.05) with the hull-less trait (Table 4 and Table S2).

**Figure 3:**
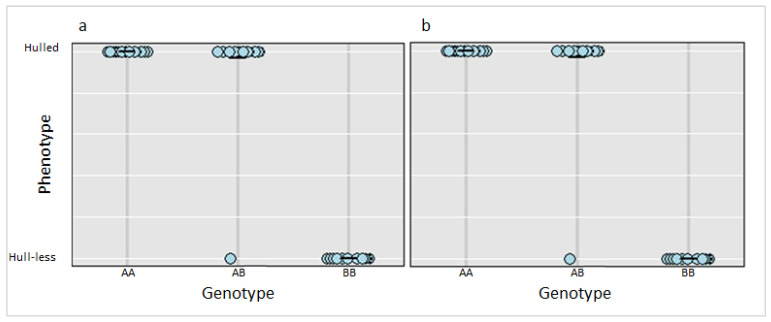
Genotype plot for SNP markers (**a**) Ch12_3412046 and (**b**) Ch12_3417142 in the F_2_ population (*n* = 143) and the corresponding phenotypic distribution. AA, AB and BB represent homozygous state for hulled phenotype, heterozygous state for hulled phenotype and homozygous state for hull-less phenotype, respectively. Each dot on the plot represents an F_2_ individual in the population.

Scheme 12. 3412046 and Ch12_3417142 were tested in the entire F_2_ population and confirmed to be significantly linked to the hull-less trait in *C. pepo (p*-value 0.00002) (Figure 3).

A scan of *Qtlhull-less-C12* interval revealed 363 genes, two among which have predicted involvement in cellulose and lignin biosynthesis in plants. The gene Cp4.1LG12g04350 (3,411,999–3,413,380) is a NAC domain-containing protein with a > 80% homology to the WOOD-ASSOCIATED NAC DOMAIN protein 3 (PdWND3A) in Populus (*Populus deltoides*). On the other hand, Cp4.1LG12g04470 (3,712,591–3,716,476) is a Fiber protein fb11 gene homologous to AL_TUS_1279 unigene protein in Monterey pine (*Pinus radiata*). Interestingly, SNP marker Ch12_3412046 lies within the first exon of Cp4.1LG12g04350 (C/T: hulled/hull-less). On the other hand, SNP marker Ch12_3417142 (A/C: hulled/hull-less) is 3.7 Kb downstream of Cp4.1LG12g04350 and 295 Kb upstream of Cp4.1LG12g04470.

## 4. Discussion

Efficient breeding for the hull-less seed trait in *C. pepo* requires a robust high throughput assay for MAS. Although the visual selection for the trait is easy, it is resource intensive as it requires the development of large, advanced segregating populations and rogueing out of undesirable individuals in the breeding program [10]. Previous mapping work in *C. pepo* helped to identify the location for the trait on linkage group LGp9 [15], but high-throughput assays for the SSR markers adjacent to the locus are costly [16]. SNP-based KASP assays [23] are a suitable alternative as they offer less handling time and allow high throughput genotyping with reduced error rate [32]. In the current study, the QTL-seq approach [19] was used to saturate the genomic location for the hull-less seed trait in *C. pepo* with SNP markers and develop KASP markers for high throughput genotyping and MAS in squash breeding.

The phenotypic ratio of 3:1 (hulled: hull-less) observed in F_2_ population supports previous reports that a single recessive gene (*h*) controls the hull-less seed trait in *C. pepo* [11,12]. In the current study, a single QTL (*Qtlhull-less-C12*) associated with the hull-less seed phenotype in Kakai was detected on chromosome 12 of the *C. pepo* genome. *Qtlhull-less-C12* corresponds to the *h* locus previously mapped in Lady Godiva (hull-less) cultivar on linkage group LGp9 further validating the position of the QTL [15]. The SSR marker (CMTm239-CAAAGATCTGTTGTGTCAGAGT) closest to the *h* locus on LGp9 extends from 4,753,918–4,753,939 bp on Chromosome 12 and maps within the *Qtlhull-less-C12* interval [15]. Among the markers tested within *Qtlhull-less-C12*, twelve were significantly associated with the hull-less trait in the F_2_ bulks. However, ten of these markers were not useful when tested among landraces and cultivars within subspecies pepo. This was expected because of the low genetic diversity and marker polymorphism at the subspecies level in *C. pepo* [33]. Further analysis identified two SNP markers (Ch12_3412046 and Ch12_3417142) that were polymorphic within subspecies pepo and could distinguish among cultivars and accessions of diverse genetic backgrounds. Surprisingly, one F_2_ individual (SS1135-1-65; Figure 1) exhibited hull-less seed phenotype, despite having a AB genotype (Figure 3). This may imply that other potential modifying loci not captured in the current study may play a role in seed coat formation. Indeed, we observed minor variation in the degree of seed coat covering among the F_2_ individuals with hull-less seed phenotype.

The SNP marker Ch12_3412046 lies within Cp4.1LG12g04350, a NAC domain-containing protein homologous to the WOOD-ASSOCIATED NAC DOMAIN protein 3 (PdWND3A) in Populus [34]. Mutants of Populus overexpressing PdWND3A were reported to have a significantly higher amount of lignin content when compared to the wild type [34]. In Castor bean (*Ricinus communis* L.), NAC domain-containing genes were reportedly overexpressed in seed tissues of genotypes with greater accumulation of lignin [35]. If Cp4.1LG12g04350 contributes to lignin accumulation in pumpkin seed coat, we would expect higher expression of the gene in the hulled parent. However, to the contrary, Wyatt et al. [17] reported that Cp4.1LG12g04350 transcripts levels were significantly higher in the hull-less cultivar (Lady Godiva) when compared to the hulled parent (Sweet REBA). Therefore, it is likely that Cp4.1LG12g04350 contributes to the differential accumulation of lignin content in other fruit tissues and not the seed (PRJNA339848; http://cucurbitgenomics.org/bioproject/50, accessed on 10 February 2022). On the other hand, Cp4.1LG12g04470, is a Fiber protein fb11 gene homologous to AL_TUS_1279, a unigene in Monterey pine involved in cell wall deposition and juvenile wood density [36]. However, transcripts of Cp4.1LG12g04470 were not differentially accumulated between hull-less and hulled seed tissues across time points [17].

## 5. Conclusions

In the current study, QTL-seq was employed to identify a QTL (*Qtlhull-less-C12*) and SNP markers associated with the hull-less seed trait in *C. pepo*. Among the twenty-eight SNP markers tested, two (Ch12_3412046 and Ch12_3417142) were significantly associated with the hull-less seed trait in the F_2_ population and validated in a set of landraces and cultivars of diverse genetic backgrounds. The KASP markers developed and validated in the current study will facilitate MAS for the hull-less seed trait in pumpkin.

## Figures and Tables

**Table 1:** Chi-square test for the inheritance of hull-less seed trait in the F_2_ population between Kakai (hull-less) and Table Gold Acorn (TGA, hulled).

Genotype	Hulled	Hull-Less	Expected Ratio (Hulled: Hull-Less)	χ^2^	*p* Value
Kakai	0	12	-	-	
Table Gold Acorn	12	0	-	-	
F_1_: Kakai × TGA	12	0	-	-	
F_2_: Kakai × TGA	112	31	3:1	0.841	0.3590 (NS)

NS = χ^2^ value not significant (*p*-value > 0.05), accept the expected hypothesis of 3:1 ratio.

**Table 2:** Whole genome resequencing statistics for hull-less and hulled parents and the bulks used in the study.

Genotype	Mapping Rate (%)	Sequencing Depth
Kakai	98.91	57.78
Table Gold Acorn	97.24	68.79
Hulled bulk	95.31	67.71
Hull-less bulk	97.81	70.26

**Table 3:** Chromosomal (Chromosome 12 (Ch12)) position, *p*-values and LOD scores of SNP markers tested for association with hull-less seed trait in the individuals constituting the bulks in the F_2_ population between Kakai (hull-less) and Table Gold Acorn (hulled).

Marker	Genomic Position (bp) ^1^	Mutation	*p*-Value *	LOD *
Ch12_2976476	2976476	T/C	0.0006	4.72
Ch12_3412046	3412046	C/T	0.0006	4.72
Ch12_3412133	3412133	C/T	0.0006	4.72
Ch12_3412203	3412203	C/T	0.002	4.70
Ch12_3412341	3412341	T/C	0.0006	4.72
Ch12_3417142	3417142	A/C	0.0006	4.72
Ch12_4323103	4323103	A/G	0.0001	6.01
Ch12_4326161	4326161	C/T	0.0001	6.01
Ch12_4326524	4326524	A/G	0.0001	6.01
Ch12_4327827	4327827	C/T	0.04	3.20
Ch12_4328336	4328336	T/C	0.03	3.04
Ch12 _4382130	4382130	C/T	0.0001	5.99

* Significant association of markers with hull-less seed trait (*p* < 0.05) for Kruskal–Wallis test and 99% for LOD scores; ^1^ Position of marker in *Cucurbita pepo* genome.

**Table 4:** Chromosomal position and Kruskal–Wallis test *p*-values of SNP markers tested for association with hull-less seed trait in cultivars and land races of diverse genetic backgrounds.

Marker	Genomic Position (bp) ^1^	*p*-Value *
Ch12_2976476	2976476	0.31
Ch12_3412046	3412046	0.008 *
Ch12_3412344	3412344	0.31
Ch12_3412203	3412203	0.31
Ch12_3412341	3412341	0.80
Ch12_3417142	3417142	0.002 *
Ch12_4323103	4323103	0.22
Ch12_4326161	4326161	0.08
Ch12_4326524	4326524	0.056
Ch12_4327827	4327827	0.06
Ch12_4328336	4328336	0.09
Ch12 _4382130	4382130	0.09

* Significant association of markers with hull-less seed trait (*p* < 0.05); ^1^ Position of marker in *Cucurbita pepo* genome.

## Data Availability

The data presented in this study are available on request from the corresponding author. The data are not publicly available due to pursuance of functional markers and potential intellectual property.

## Supplementary Materials

The following are available online at https://www.mdpi.com/article/10.3390/plants11091238/s1, Table S1: Sequence and genomic location for the twenty-eight SNP markers assayed in the F_2_ population between Kakai (hull-less) and Table Gold Acorn (hulled). Table S2: SNP marker genotype for two KASP markers (Ch12_3412046 and Ch12_3417142) and corresponding seed phenotype for Cucurbita pepo accessions assayed in the study. Figure S1: Fruit (a) and seeds (b) of Kakai (hull-less) and Table Gold Acorn (hulled) cultivars used to generate the F_2_ mapping population in the study. Figure S2: Quantitative trait loci (QTL) associated with hull-less seed phenotype in *Cucurbita pepo* on chromosome 12 using Table Gold Acorn as the consensus reference genome. Blue, red and green lines represent 90, 95 and 99% confidence interval (CI), respectively. Figure S3: Quantitative trait loci (QTL) associated with hull-less seed phenotype in *Cucurbita pepo* on chromosome 12 using Kakai as the consensus reference genome. Red, green and blue lines represent 90, 95 and 99% confidence interval (CI), respectively. Figure S4: Logarithm of odds (LOD) scores for the twelve SNP markers genotyped in the individuals constituting the hulled and hull-less bulks. The dotted green line represent 99% confidence interval.
