# Supplementary figures and images for: Genomic Position and Markers Associated with the Hull-Less Seed Trait in Pumpkin
Source: Plants (Basel). 2022 May 3;11(9):1238. doi: 10.3390/plants11091238 (PMC9103792; doi:10.3390/plants11091238)

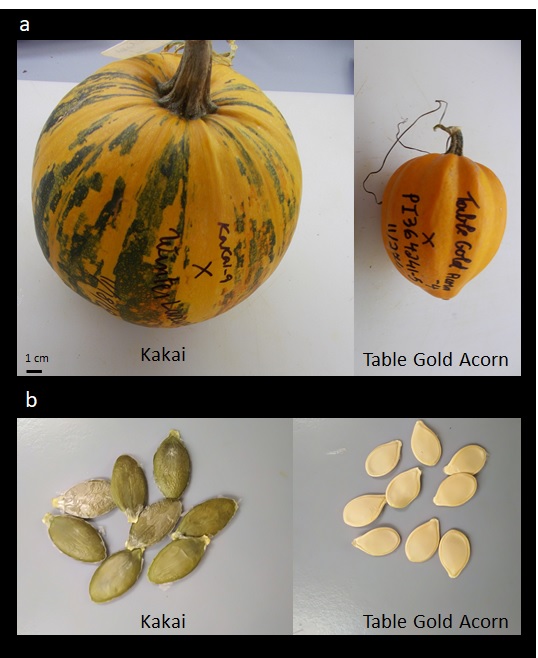

Supplement: Supplementary file 1 [file plants-11-01238-s001.zip › Figure S1.jpg]

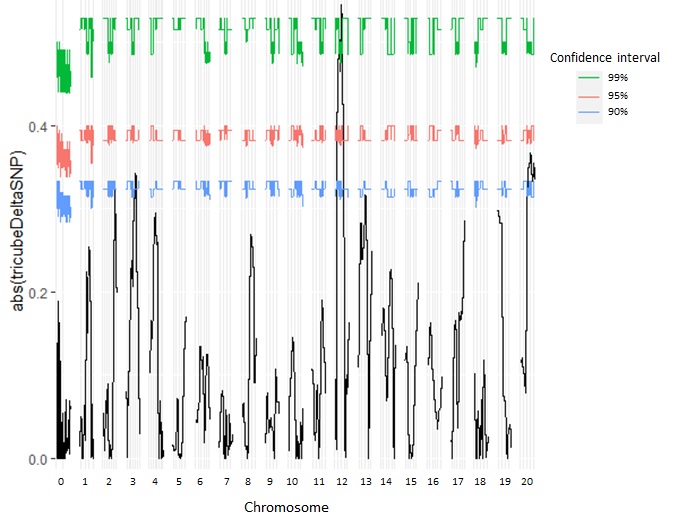

Supplement: Supplementary file 1 [file plants-11-01238-s001.zip › Figure S2.jpg]

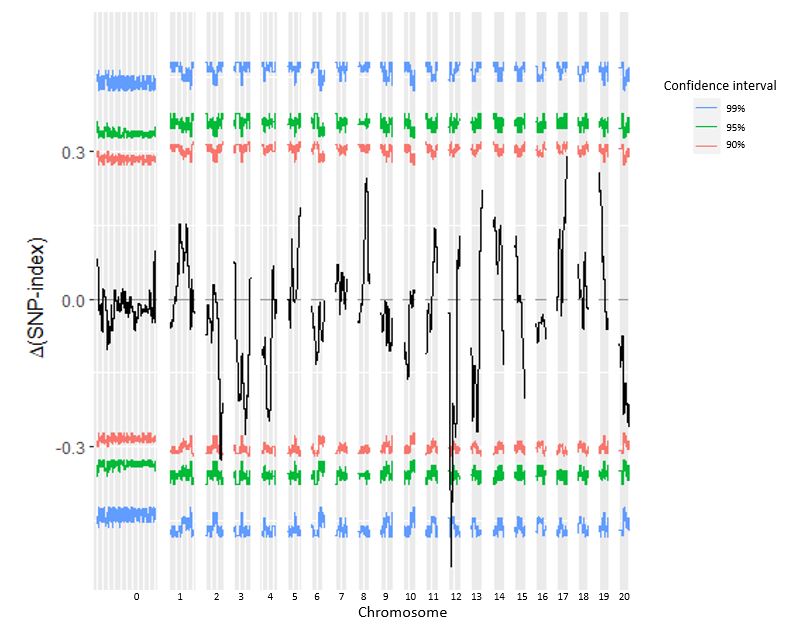

Supplement: Supplementary file 1 [file plants-11-01238-s001.zip › Figure S3.JPG]

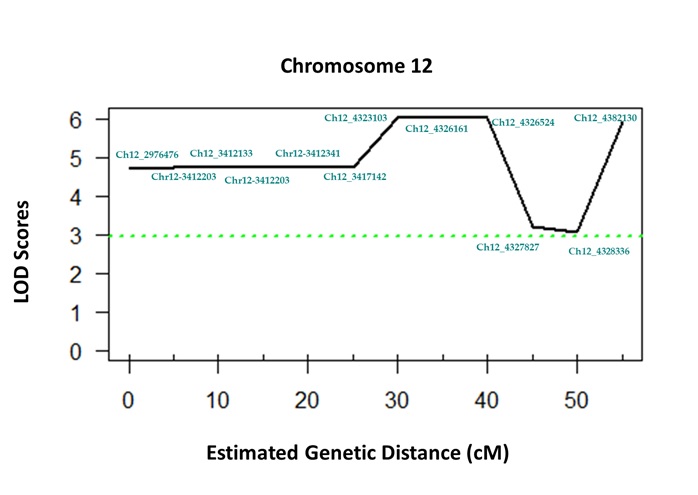

Supplement: Supplementary file 1 [file plants-11-01238-s001.zip › Figure S4.jpg]
